# Quinoline-Malononitrile-Based Aggregation-Induced Emission Probe for Monoamine Oxidase Detection in Living Cells

**DOI:** 10.3390/molecules28062655

**Published:** 2023-03-15

**Authors:** Chuthamat Duangkamol, Sirilak Wangngae, Sirawit Wet-osot, Onnicha Khaikate, Kantapat Chansaenpak, Rung-Yi Lai, Anyanee Kamkaew

**Affiliations:** 1 School of Chemistry, Institute of Science, Suranaree University of Technology, Nakhon Ratchasima 30000, Thailand; 2 Division of Basic and Medical Sciences, Faculty of Allied Health Sciences, Pathumthani University, Pathum Thani 12000, Thailand; 3 Medical Life Science Institute, Department of Medical Sciences, Ministry of Public Health, Nonthaburi 11000, Thailand; 4 National Nanotechnology Center, National Science and Technology Development Agency, Thailand Science Park, Pathum Thani 12120, Thailand

**Keywords:** AIE, activity-based fluorescence probe, MAO, enzymatic detection, AIEgen

## Abstract

A quinoline-malononitrile (QM)-based aggregation-induced emission probe was developed to detect MAOs in cells through an enzymatic reaction followed by β-elimination. After being incubated at 37 °C, **QM-NH_2_** responded to the MAO enzymes with great specificity and within just 5 min. This 5 min responsive mechanism was fast, with the limit of detection (LOD) at 5.49 and 4.76 µg mL^−1^ for MAO-A and MAO-B, respectively. Moreover, **QM-NH_2_** displayed high enzyme specificity even in the presence of high concentrations of biological interferences, such as oxidizing and reducing agents, biothiols, amino acids, and glucose. Furthermore, **QM-NH_2_** demonstrated biocompatibility as the cells retained more than 70% viability when exposed to **QM-NH_2_** at concentrations of up to 20 µM. As a result, **QM-NH_2_** was used to detect MAO-A and MAO-B in SH-SY5Y and HepG2 cells, respectively. After 1h incubation with **QM-NH_2_**, the cells exhibited enhanced fluorescence by about 20-fold. Moreover, the signal from cells was reduced when MAO inhibitors were applied prior to incubating with **QM-NH_2_**. Therefore, our research recommends using a QM probe as a generic method for producing recognition moieties for fluorogenic enzyme probes.

## 1. Introduction

Monoamine oxidases A and B (MAO-A and MAO-B) have attracted considerable interest from the pharmaceutical and biochemical communities since 50 years ago [1,2,3,4]. For instance, malonic acid’s neurodegenerative toxicity and striatal damage can be considerably reduced by MAO inhibition, whereas MAO overexpression can lead to mitochondrial damage and an imbalance in oxidoreduction, which can result in neuronal death and brain damage [5]. MAOs are a flavoprotein that plays an important role in regulating tissue that catalyzes oxidation of monoamine to form the corresponding imine, followed by hydrolysis to an aldehyde [6]. There are specific inhibitors, clorgyline for MAO-A and pargyline for MAO-B [7]. MAO-A is a key target for being a primary cause of neuropsychiatric and depressive disorders, whereas MAO-B is strongly associated with a number of neurodegenerative diseases [8,9,10,11]. The specific detection of each MAO is thus critical for a better understanding of their functions in complex biosystems.

Quinoline-malononitrile (QM) was introduced to the original family as an aggregation-induced emission (AIE) building block, which is extremely helpful in understanding the molecular structure–property relationship [12]. Guo et al. developed an emitter named ED with an impressive alternative approach to modify the typical π-electron acceptor in dicyanomethylene-4*H*-pyran chromophore (DCM) derivatives [13]. Many DCM derivatives have previously been reported due to their unique intramolecular charge transfer (ICT) and excellent opto-electronic properties. In general, the DCM chromophore has the typical Donor-π-Acceptor (D-π-A) property with a broad absorption band as a result of an ultra-fast ICT process [14]. However, due to the severe aggregation-caused quenching (ACQ) effect, conventional DCM derivatives can only be used as dopant emitters [15,16]. Strong face-to-face stacking with a typical ACQ effect occurs in the solid state as a result of the strong π–π interaction between the aromatic rings of DCM. On the other hand, the addition of an *N*-ethyl group to the acceptor of QM effectively suppresses the close stacking of molecules in its aggregate process [12]. Because of the aqueous physiological environment, water-soluble AIE systems are highly desirable for tracing important biological species.

**Figure 1:**
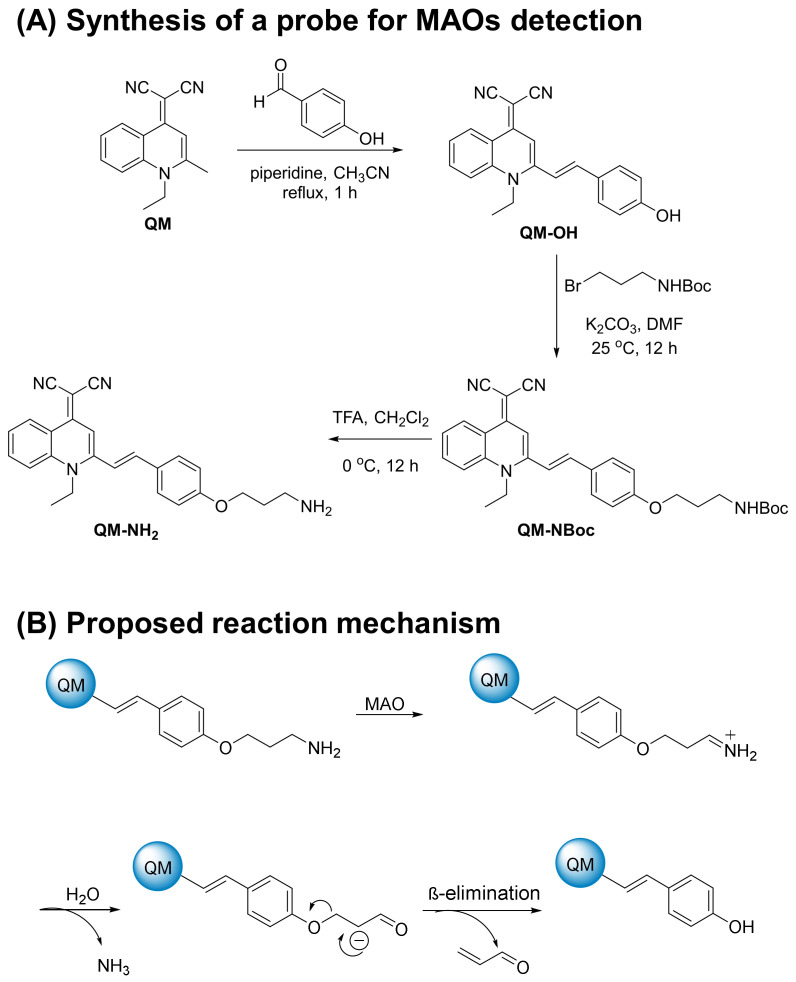
(**A**) Synthetic scheme of **QM-NH_2_**. (**B**) Proposed reaction mechanism of **QM-OH** generation from **QM-NH_2_**. MAOs oxidize the amine of **QM-NH_2_** to form the imine followed by hydrolysis to generate the aldehyde. Finally, the aldehyde undergoes β-elimination to generate **QM-OH**.

Fluorescent materials are now crucial for biological investigation and clinical diagnosis since they offer dynamic and quantitative information about imaging biomolecules [17,18,19]. Because AIE materials exhibit turn-on fluorescence in aggregation while dim in solution [20], the fascinating AIE phenomenon in particular offers prospective advantages, especially in biosensing applications [21,22,23,24]. Due to its great sensitivity, the use of AIE fluorescent probes in conjunction with confocal imaging techniques is increasingly becoming more and more common for monitoring MAOs in living biosystems [25,26,27]. The existing fluorescence probes are made to detect either MAO-A [25,26,27] or MAO-B [28,29] with AIE properties. For example, the methyl substituent on tetraphenylethylene (TPE) derivatives (*trans*-TPEM), among the six produced probes, exhibits a notable increase in fluorescence when MAOs are added with selectivity toward MAO-A [30]. In a different work, TPE was synthesized with an extended conjugation to thiophene to create TPE-TPys, which had a perfect D–π–A structure and a high level of positive charges [31]. These compounds demonstrated outstanding MAO-A specificity, high-efficiency photosensitivity, and mitochondrial targeting. To the best of our knowledge, none of the QM-based MAO fluorescent turn-on probes are AIE-active probes [4,30]. As a result, it would be interesting to investigate the AIE probe’s ability to detect MAOs through the reaction-based sensing scheme (Figure 1B).

In this study, we developed an AIE probe based on the QM building block, **QM-NH_2_**, which has reduced fluorescence and may be used to detect MAOs in cells since it has a responsive moiety consisting of propylamine. When the probe binds to MAOs after oxidation and hydrolysis, it undergoes β-elimination, releasing **QM-OH**, an AIE agent that activates the fluorescence signal. Our probe provided a fast response to MAO activity, within 5 min, in both solution and living cells. It was also available for real-time and in situ imaging with good photostability, high resolution, and long-term tracking capabilities. We believe that our **QM-NH_2_** overcomes the inherent shortcomings of ACQ fluorophores and offers better properties than the conventional AIEgens, even though the majority of available AIE probes always emphasize the improper aggregation in both the aqueous system and lipophilic organelle before binding to the specific receptor.

## 2. Results and Discussions

### 2.1. Synthesis

The synthetic steps of an enzyme-activatable AIE-active probe (**QM-NH_2_**) based on AIEgen **QM-OH** with a *para*-phenolic group protected by propylamine, an MAO-triggered unit, are described in Figure 1A. Starting with an AIE building block, quinoline-malononitrile (**QM**), a signal reporter, was prepared from *N*-methylated 2-methylquinoline and propane dinitrile [31]. Then, **QM** was condensed with the 4-hydroxybenzaldehyde in the presence of piperidine under the reflux condition to obtain the AIEgen **QM-OH** [32] in a moderate yield, 57%. After that, **QM-OH** was directly conjugated with the *t*-Boc-protected primary bromopropylamine to produce the compound **QM-NBoc** in 68% yield. Finally, the deprotection of **QM-NBoc** with trifluoroacetic acid at low temperature produced the amino-ended **QM-NH_2_** at 56% yield as an orange solid. Detailed synthetic procedure (Scheme S1) and characterization (^1^H-NMR, ^13^C-NMR, and HRMS) are depicted in the Supplementary Materials.

The general concept of fluorescence enzyme assays is that compounds are not fluorescence substrates until they are acted on by their enzymes of interest. The amount of light produced by the reaction of **QM-NH_2_** with the liberated substrate, most commonly **QM-OH**, can then be linked to the activity of the enzyme of interest. Because MAO oxidizes primary, secondary, and tertiary acyclic amines to produce iminium intermediates, which are hydrolyzed to the corresponding aldehydes (Figure 1B), we reasoned that such iminium and/or aldehyde derivatives could aid in the liberation of **QM-OH** via a β-elimination process.

### 2.2. Photophysical Properties

**Figure 2:**
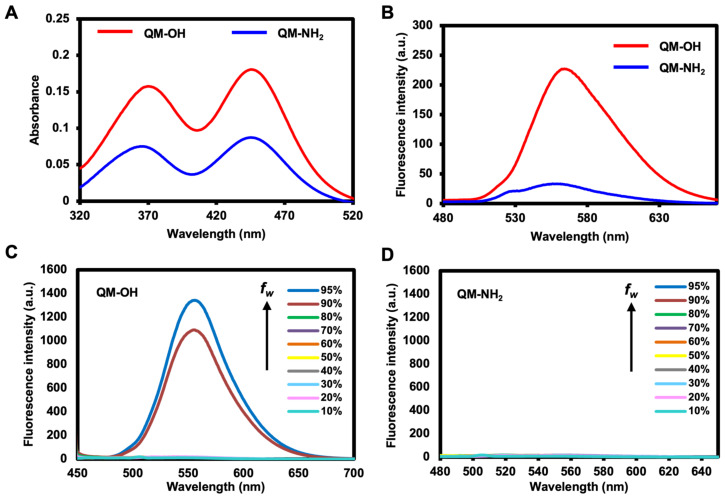
(**A**) Absorption spectra of **QM-NH_2_** and **QM-OH** in HEPES buffer. (**B**) Emission spectra (excited λ = 445 nm) of **QM-NH_2_** and **QM-OH** in HEPES buffer. Aggregation-induced emission property of (**C**) **QM-OH** and (**D**) **QM-NH_2_** investigated in tetrahydrofuran (THF)–water mixtures with different water fractions (*f_w_*) in which THF is a good solvent and water is a poor solvent.

**QM-NH_2_** and **QM-OH** (the proposed product after the MAO enzymatic reaction) were tested for their photophysical properties. Every compound has an absorbance in HEPES buffer (pH 7.4) that ranges from 300–550 nm with two peaks at 370 and 450 nm, which is characteristic of QM (Figure 2A). Compared to **QM-NH_2_**, **QM-OH** has a substantially greater emission (8-fold higher) in HEPES buffer, as shown by the emission spectrum, peaking at 559 nm (Figure 2B) with a large Stokes shift of 114 nm. Fluorescence quantum yields for **QM-NH_2_** and **QM-OH** in the buffer are calculated as 0.003 and 0.025, respectively, confirming high fluorescent emissions for **QM-OH**. Then, the aggregation-induced emission (AIE) property of **QM-OH** was investigated in tetrahydrofuran (THF)–water mixtures with different water fractions (*f_w_*). As shown in Figure 2C, the emission intensity changes of **QM-OH** were negligible as *f_w_* was increased from 0% to 80%. When the *f_w_* reached 90%, the fluorescence intensity increased sharply by about 360-fold from pure THF, indicating the strong AIE property of **QM-OH** in the medium with high water contents. On the other hand, the **QM-NH_2_** probe exhibited non-emissive reactions in all water fractions as demonstrated in Figure 2D. These results suggest that **QM-NH_2_** could potentially detect MAO through the fluorescence enhancement phenomenon in the aggregated form.

#### 2.2.1. Fluorescence Responses of **QM-NH_2_** to MAOs

**Figure 3:**
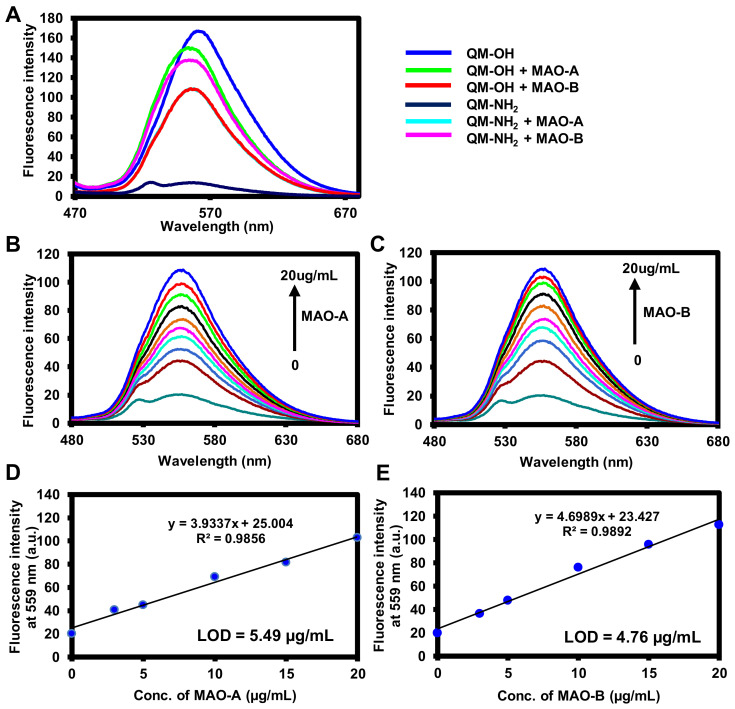
(**A**) Fluorescence spectra of 10 μM **QM-NH_2_** (navy blue line) and **QM-NH_2_** after reaction with 20 μg/mL of MAO-A (cyan line) and MAO-B (red line). The synthesized **QM-OH** (10 μM, blue line) and its reaction with 20 μg/mL of MAO-A (green line) and MAO-B (pink line) were tested as the controls. Fluorescence responses of **QM-NH_2_** reacted with (**B**) MAO-A and (**C**) MAO-B at concentrations ranging from 0–20 µg/mL. The data were recorded in the enzymatic assay buffer (100 mM HEPES, pH = 7.4 with 5% glycerol and 1% DMSO) at 37 °C after 5 min incubation. Linear fluorescence intensity responses of **QM-NH_2_** reacting with various concentrations of (**D**) MAO-A and (**E**) MAO-B.

The response of **QM-NH_2_** for MAO-A and MAO-B was then investigated. The enzymatic reactions were performed in 100 mM HEPES buffer (pH 7.4) at 37 °C for 5 min. Figure 3A showed the fluorescent spectroscopic properties of **QM-NH_2_** and its response to MAOs. Four different scenarios were tested under the physiological condition: (i) **QM-OH** alone, (ii) **QM-OH** in the presence of MAO-A or MAO-B, (iii) **QM-NH_2_** in the presence of MAO-A or MAO-B, and (iv) **QM-NH_2_** alone. Probe **QM-NH_2_** (condition iv) exhibited almost no emission in the HEPES buffer, but when it reacted with MAOs (condition iii), a strong emission enhancement at 559 nm was observed. The emission spectra of **QM-NH_2_** in the presence of MAOs are similar to those of **QM-OH** in the presence of MAOs, implying that **QM-OH** is the product of the reactions. Furthermore, the responses of **QM-NH_2_** to MAOs are dose-dependent as the fluorescence increments were directly proportional to the enzyme concentrations (Figure 3B,C). The linear relationships between the fluorescent signals and the enzyme concentrations were observed at the 0–20 µg/mL range of MAO-A and MAO-B (Figure 3D,E). The detection limits of **QM-NH_2_** were calculated as 5.49 and 4.76 µg/mL for MAO-A and MAO-B, respectively.

#### 2.2.2. HPLC Analysis of the Enzymatic Reaction

**Figure 4:**
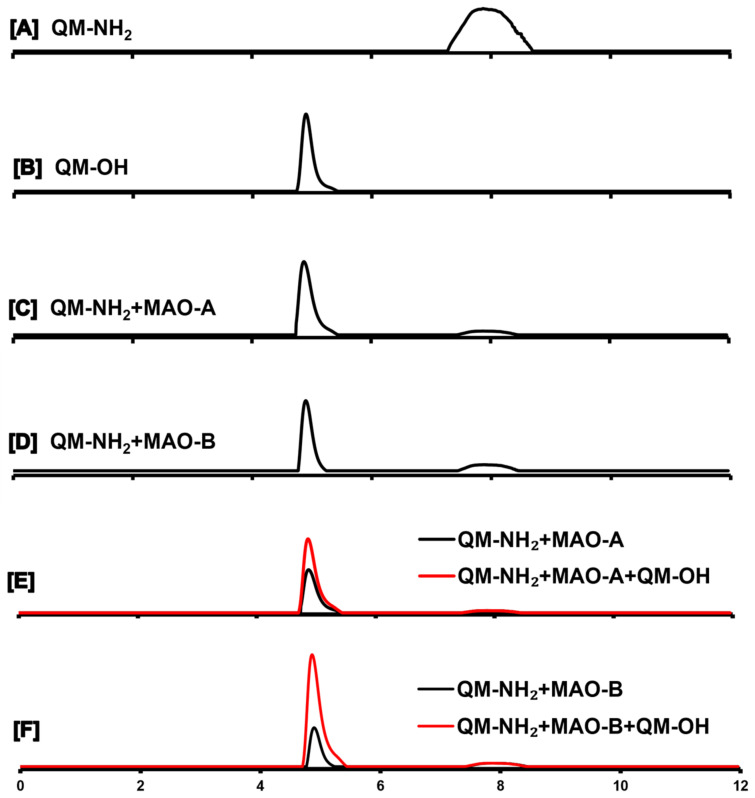
HPLC chromatograms of different reaction systems: (**A**) 100 μM **QM-NH_2_**; (**B**) 100 μM **QM-OH**; (**C**) the reaction of 100 μM **QM-NH_2_** treated with 20 µg/mL MAO-A for 5 min; (**D**) the reaction of 100 μM **QM-NH_2_** treated with 20 µg/mL MAO-B for 5 min; (**E**) comigration of **QM-NH_2_** + MAO-A reaction with **QM-OH** spiking; and (**F**) comigration of **QM-NH_2_** + MAO-B reaction with **QM-OH** spiking. Detection: UV–vis (400 nm) detector. Flow rate: 0.7 mL/min. Temperature: 25 °C. Injection volume: 40 μL. Mobile phase (isocratic elution): MeOH/water = 70/30 (*v*/*v*) for 12 min.

To identify the product of **QM-NH_2_** after MAO-catalyzed reaction, the reaction mixtures of MAO-A and MAO-B were subjected to HPLC analysis as shown in Figure 4. The chromatograms of **QM-NH_2_** and **QM-OH** are displayed in Figure 4A,B, while the ones for the MAO-A- and MAO-B-catalyzed reactions in the existence of **QM-NH_2_** are demonstrated in Figure 4C,D, respectively. The presence of the peak at 4.89 min retention time suggested the formation of **QM-OH** after the enzymatic reactions. To further confirm the peak identity, the standard of **QM-OH** was spiked into each reaction mixture to show the comigration as manifested in Figure 4E,F. Furthermore, the eluent of each peak was collected for mass spectrometric analysis to confirm its identity. According to HPLC and mass spectrometric analyses, it was proven that the enzymes (MAO-A and MAO-B) oxidized **QM-NH_2_**, followed by hydrolysis and β-elimination to generate **QM-OH**.

#### 2.2.3. Selectivity of **QM-NH_2_** toward MAOs

**Figure 5:**
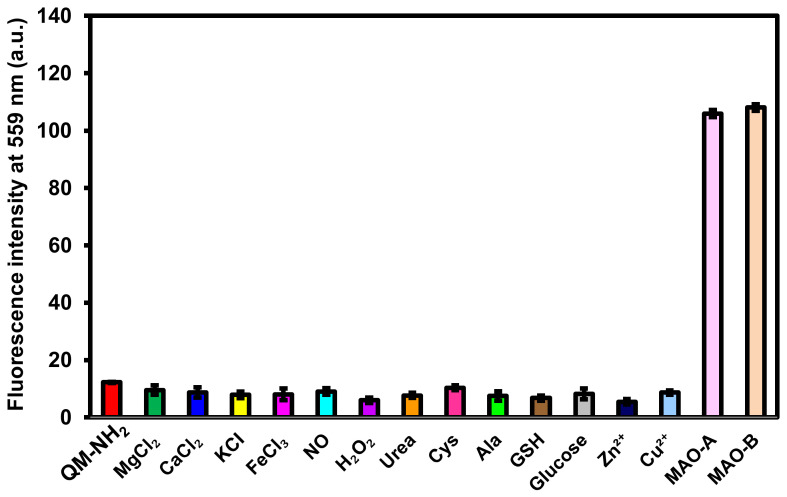
Fluorescence response of **QM-NH_2_** (10 μM) after 5 min of incubation at 37 °C with various analytes: MgCl_2_ (2.5 mM), CaCl_2_ (2.5 mM), KCl (150 mM), FeCl_3_ (100 µM), NO (100 µM), H_2_O_2_ (100 µM), urea (20 mM), cysteine (1 mM), alanine (1 mM), reducing glutathione (GSH, 1 mM), glucose (10 mM), Zn^2+^ (1 mM), Cu^2+^ (1 mM), MAO-A (20 μg/mL), and MAO-B (20 μg/mL). The results are expressed as the mean SD (n = 3). λ_ex_/_em_ = 455/559 nm.

To investigate the selectivity of **QM-NH_2_**, the probe was incubated with cations, anions, urea, amino acids, or glutathione to observe if the fluorescence turned on or not. As expected, the fluorescence intensities at 559 nm produced by MAO-A and MAO-B are significantly higher than that produced by other analysts, as shown in Figure 5. Furthermore, when **QM-NH_2_** was exposed to MAOs at high concentrations of oxidizable interferences, such as amino acids (alanine and cysteine) and glucose, the fluorescence intensity did not change significantly (Figure S1). According to these findings, the probe **QM-NH_2_** has a high selectivity for MAOs.

Following that, we investigated the effects of temperature factors on fluorescence intensity at 559 nm. As seen in Figure S2, the fluorescence changes upon the addition of enzymes increased along with the enhancement of reaction temperatures from 25 to 37 °C as the enzyme activity increased. However, at 45 °C, the fluorescence change dropped, which could be the result of enzyme denaturation at high temperatures. Therefore, the optimum temperature for these enzymatic reactions is 37 °C, which is a physiological condition. Next, we tested the stability of probes **QM-NH_2_** and **QM-OH** by incubating the probes in HEPES buffer, pH = 7.4. According to Figure S3, the probes **QM-NH_2_** and **QM-OH** in HEPES buffer only showed a slight change in fluorescence signal, indicating that probe **QM-NH_2_** and **QM-OH** were stable in the solution for at least 48 h. These results demonstrated that determining MAOs using the probe **QM-NH_2_** was suitable for biological applications advantageous in physiological conditions.

#### 2.2.4. Interactions of **QM-NH_2_** and MAOs Visualized by Molecular Docking

**Figure 6:**
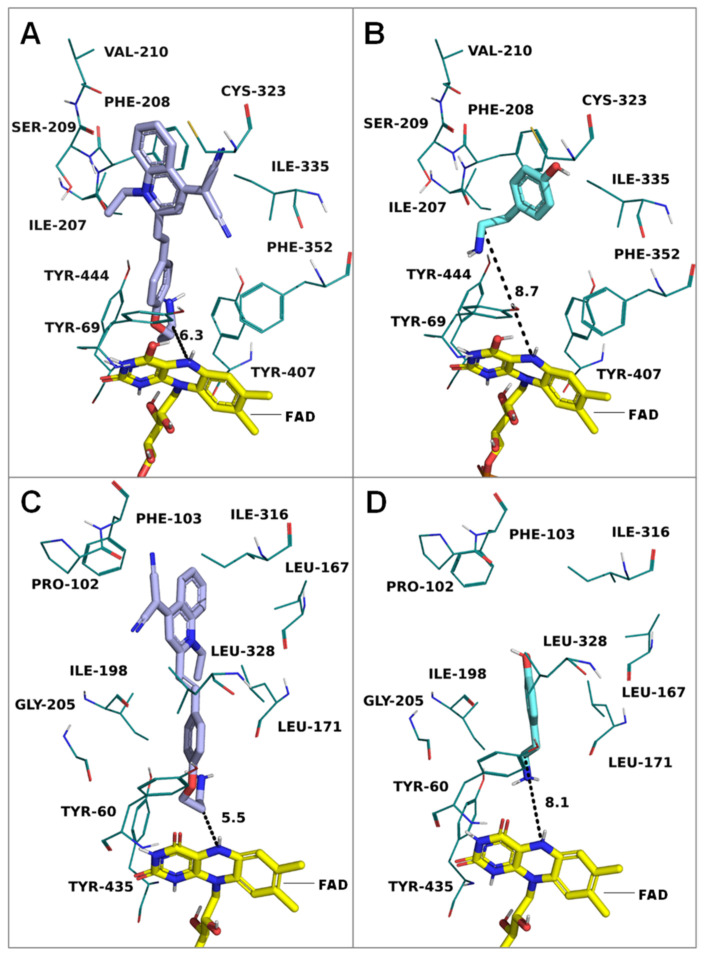
Calculated docking poses of **QM-NH_2_** and tyramine in the active site of MAO-A (PDB: 2BXR) and MAO–B (PDB: 2V5Z) crystal structures: (**A**,**B**) docking poses of **QM-NH_2_** and tyramine with MAO-A, respectively; (**C**,**D**) docking poses of **QM-NH_2_**, and tyramine with MAO-B, respectively. FAD is shown in yellow sticks; important residues of MAO are depicted as deep cyan lines, and distance measurements are shown in dashed lines.

Molecular docking is a powerful tool for describing the interactions between a compound and a protein receptor. In silico docking of **QM-NH_2_** with MAO-A and MAO-B was used to estimate the preferred orientation of the compound inside the active site using AutoDock 4.2. The computational results showed that **QM-NH_2_** fits both MAO-A and MAO-B in their active site regions (MAO-A: Tyr69, Ala111, Ile207, Phe208, Val210, Gln215, Cys323, Ile335, Phe352, Tyr444) (Figure 6A) and (MAO-B: Tyr60, Pro102, Phe103, Leu167, Leu171, Ile198, Gly205, Gln206, Ile316, Tyr326, Leu328, Tyr435) (Figure 6C). The distances of the carbon adjacent to the terminal amine and the N5 atom of FAD are 6.3 Å in MAO-A and 5.5 Å in MAO-B, respectively, suggesting that **QM-NH_2_** was oriented in the direction of FAD in close proximity [33]. Additionally, to verify the used docking method, the docking comparative experiments were performed using tyramine, a non-specific inhibitor of both MAO isozymes (Figure 6B,D). The results showed that **QM-NH_2_** could bind to both MAO-A and MAO–B in a similar orientation to tyramine. The binding free energy calculations and residue contacts are summarized in Table S1.

### 2.3. Cellular Assays

**Figure 7:**
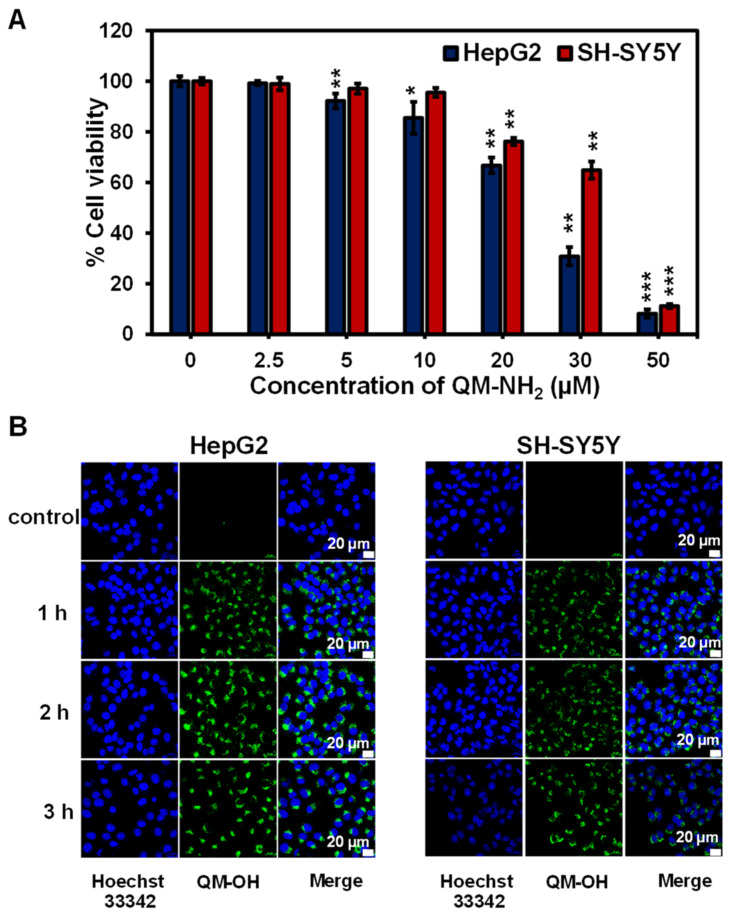
(**A**) Relative cell viability of SH-SY5Y and HepG2 cells treated with **QM-NH_2_** at different concentrations for 24 h. Statistical analysis is based on T-test (* *p* < 0.05, ** *p* < 0.01, *** *p* < 0.001). (**B**) Confocal images of time-dependent enzymatic reactions for the detection of **QM-OH** in SH-SY5Y and HepG2 cells were obtained using a laser scanning confocal microscope (Nikon A1Rsi, 63× oil immersed optics). The cells were incubated with 10 μM of **QM-NH_2_** for 1–3 h, nucleus is shown in blue as Hoechst 33342 signal (excitation laser = 405 nm, emission band 450/25 nm); **QM-OH** can be detected as green fluorescence (excitation laser = 488 nm, emission band 515/30 nm).

Next, the biocompatibility of **QM-NH_2_** was tested in two cancer cell lines, human neuroblastoma (SH-SY5Y) and human liver (HepG2) cells. The cells were treated with various concentrations of **QM-NH_2_** (0, 2.5, 5, 10, 20, 30, and 50 μM) for 24 h. The comparative cell viability was determined by the standard MTT assay [34]. According to Figure 7A, when SH-SY5Y cells, which overexpress MAO-A [35], and HepG2 cells, which overexpress MAO-B [36], were exposed to **QM-NH_2_**, the cell viability was still greater than 70%. Higher concentrations, i.e., 30–50 µM, however, caused the cell viability to start declining quickly. This might be because of the aggregation at high concentrations. As a result, it is safe to apply doses in the 1 to 10 µM range in cellular tests.

Subsequently, the capability of **QM-NH_2_** for fluorescence imaging of endogenous MAO-A and MAO-B activity in SH-SY5Yand HepG2 cells was investigated. Time-dependent intracellular enzyme activity was performed to track the fluorescence signal of **QM-OH** generated from the MAOs-catalyzed reaction. Within a few minutes, the signal was visible from the cells, and as time increased, the fluorescence enhanced (Video in Data Availability Statement). For live cell imaging, we captured the green fluorescence observed from both cell types and the signals grew stronger over time (Figure 7B and Figure S4). Furthermore, the cancer cells fluoresced brighter in the first hour compared to the healthy cell, human foreskin fibroblasts (HFF) (Figure S5), which corresponds to the higher MAO levels expressed in cancer cells compared to normal cells [37]. As the reaction time was extended, it appeared that both malignancy and normal cells glowed brightly (Figure S5), suggesting that the ideal detection time is within the first hour.

To broaden the application of **QM-NH_2_** in the detection of MAOs in various cancer cell types, including cervical (HeLa), lung (A549), and breast (MCF-7) cells, the cells were exposed to the probe for 3 h before being fixed and imaged. Bright green fluorescence was observed from all the cells, implying that **QM-NH_2_** is compatible with all cancer types. In addition, it is intriguing that the light is still discernible after cell fixation, suggesting the potential application in immunostaining (Figure S6).

**Figure 8:**
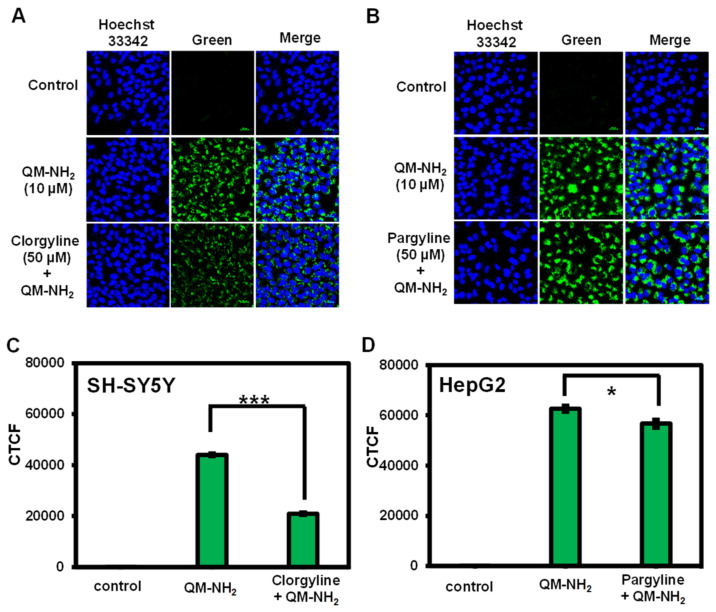
Confocal images of (**A**) SH-SY5Y and (**B**) HepG2 cells treated with **QM-NH_2_** in the absence and presence of potent MAO inhibitors (MAO-A: clorgyline and MAO-B: pargyline). (**C**,**D**) Quantitative fluorescent intensity of images in (**A**,**B**), respectively, represented as corrected total cell fluorescence (CTCF), which were quantified using ImageJ and represent the mean ± SD (from three independent experiments, 30 cells/set, respectively. Scale bar = 20 μm. Statistical analysis is based on T-test (* *p* < 0.05, *** *p* < 0.001).

Next, to confirm the selectivity toward MAO, the cells were pretreated with MAO inhibitors, clorgyline, and pargyline, before incubating with **QM-NH_2_**. As shown in Figure 8, clorgyline suppressed endogenous MAO-A activity in SH-SY5Y cells and reduced fluorescence signals by 52%. In contrast, pargyline suppressed MAO-B activity in HepG2 cells, resulting in a 10% drop in fluorescence when compared to cells not treated with the inhibitor. This suggested that **QM-NH_2_** could be selective to MAO in living cells.

## 3. Materials and Instruments

The absorption and fluorescence spectra were collected using a UV–vis spectrophotometer (Agilent Technologies Cary 300, Santa Clara, CA, USA) and a fluorescence spectrophotometer (PerkinElmer LS55, Liantrisant, UK). Nuclear magnetic resonance (NMR) spectroscopy (^1^H and ^13^C NMR) was performed at room temperature on a Bruker instrument (500 MHz) spectrometer (Rheinstetten, Germany) with TMS as an internal reference. Under the ESI condition, high-resolution mass spectroscopy (HRMS) spectra were recorded on a mass spectrometer. Prior to use, all glassware was oven dried. All reagents and solvents were purchased commercially (Sigma Aldrich, TCI (Tokyo, Japan), Carlo Erba (Emmendingen, Germany), Acros (Geel, Belgium), Merck (Rahway, NJ, USA)) and used without further purification. Purifications by column chromatography were carried out using silica gel for chromatography (Carlo Erba) as a stationary phase. Analytical thin layer chromatography (TLC) was performed on TLC Silica gel 60 F254 (Merck) and visualized under a UV cabinet. Human monoamine oxidase A and monoamine oxidase B were purchased from Sigma Aldrich.

### 3.1. UV–vis and Fluorescence Spectroscopic Methods

In all experiments, the stock solutions (10 mM) of **QM-OH** and **QM-NH_2_** were prepared in DMSO. The fluorescence spectra of **QM-NH_2_** (10 µM) with MAOs in 100 mM HEPES buffer (pH 7.4) were measured after 5 min incubation at 37 °C. For AIE property investigation, **QM-OH** and **QM-NH_2_** were dissolved in tetrahydrofuran (THF)–water mixtures with different water fractions (*fw*) from 0% to 95%. UV–vis absorption and fluorescence spectra were performed in a quartz cell with a 1 cm path length and recorded on a UV–vis spectrophotometer (T80+ UV–vis spectrometer, PG Instruments Ltd., Lutterworth, UK) and a spectrofluorometer (JASCO FP-8300, Japan, Tsukuba), respectively. The fluorescence spectra were recorded at λ_ex_ = 445 nm.

### 3.2. MAO Assays

Generally, in an assay volume of 50 μL, 0–20 μg of protein from microsomes containing MAO-A or MAO-B (Sigma-Aldrich, Taufkirchen, Germany) were incubated with 10 µM of the probe **QM-NH_2_** in the buffer (100 mM HEPES containing 5% glycerol and 1% DMSO, pH 7.4) at 37 °C for 5 min. The reaction mixtures were analyzed by the fluorescence spectroscopic method or high-performance liquid chromatography (HPLC). The HPLC instrument is Agilent 1100 (Waldbronn, Germany). The column is ZORBAX Eclipse XDB-C18 (4.6 mm × 150 mm, 5 μm ID).

### 3.3. Cell Culture

Human lines, neuroblastoma (SH-SY5Y), hepatocellular carcinoma (HepG2), cervical adenocarcinoma (HeLa), lung adenocarcinoma (A549), breast cancer (MCF-7), and foreskin fibroblasts (HFF) were cultured on 75 cm^3^ culture flasks in complete media according to the ATCC (American Type Culture Collection) protocols. All cells were cultured in a humidified incubator at 37 °C under 5% CO_2_.

### 3.4. Cell Viability Assay

The cells were seeded into 96-well cell culture plates at 2 × 10^4^ cells/well and incubated for 24 h for cell viability testing. Following that, cell culture media were replaced with media containing various concentrations of **QM-NH_2_** (0, 2.5, 5, 10, 20, 30, and 50 μM). Cell viability was determined using the standard MTT protocol after 24 h of incubation. In brief, the cells were washed three times with PBS before being treated for 2 h with methylthiazolyldiphenyl-tetrazolium bromide (20 μL, 0.5 mg/mL, Sigma-Aldrich). The culture media was then replaced with DMSO, and cell viability was determined using a microplate reader (BMG Labtech/SPECTROstar Nano, Offenburg, Germany) to detect the absorption of the resulting formazan at 560 nm.

### 3.5. Time-Dependent Internalization

Cells were seeded at a density of 7 × 10^3^ cells per well in an 8-well chambered coverglass with non-removable wells (Nunc Lab-Tek II Chamber Slide) and incubated for 24 h. After that, cell culture media were removed and the cells were pre-treated with 10 μM of **QM-NH_2_** solution in DMEM with 5% FBS for 1, 2, 3, 6, and 24 h. After incubation, the cells were washed three times with 0.01 M PBS and stained with 0.5 mg/mL of Hoechst 33342 containing media. The cells were then imaged under a laser scanning confocal microscope (LSCM, Nikon A1Rsi). The resulting **QM-OH** was visualized using a 488 nm laser excitation and emission filter of 515/30 nm. Hoechst 33342 was detected using an excitation laser = 405 nm and emission filter of 450/25 nm. A 60× oil immersion objective lens was used.

### 3.6. Time Course Live Cell Imaging

Approximately 1 × 10^4^ cells of HepG2 were seeded on a cell dish for 24 h. Then, 10 µM of **QM-NH_2_** solution in DMEM with 5% FBS was added to the cell dish, followed by a prompt VDO recording for 30 min by 60× oil immersion objective lens of laser scanning confocal microscope (Nikon A1Rsi) with 405 nm and 488 nm lasers.

### 3.7. Cell Fixation Assay

Approximately 1 × 10^4^ cells were seeded on an 8-well chambered coverglass for 24 h. Then, the cells were incubated with 10 μM of **QM-NH_2_** for 3 h. After incubation, the cells were washed three times with 0.01 M PBS, followed by the addition of 4% paraformaldehyde (PFA) and incubation for 20 min at room temperature. The cells were fixed on the coverslip containing DAPI mounting media. Then, the cells were visualized under 60× oil immersion objective lens by laser scanning confocal microscope (Nikon A1Rsi) with 405 nm and 488 nm lasers.

### 3.8. Inhibition Assay

At approximately 1 × 10^4^ cells/well, cells were seeded on an 8-well chambered coverglass (LabTek, Nunc) and incubated in completed media for 24 h. After that, SH-SY5Y and HepG2 cells were treated with clorgyline and pargyline (50 µM), respectively, for 1 h prior to incubation with **QM-NH_2_** (10 µM) for another 3 h. After incubation, the cells were washed three times with PBS and treated with 0.5 mg/mL of Hoechst 33342 containing media before being imaged by LCSM.

For the corrected total cell fluorescence (CTCF), an outline was drawn around each cell using ImageJ, and the circularity, area, and mean fluorescence were assessed, as well as numerous surrounding background readings. CTCF was calculated as follows:

CTCF = integrated density-(area of selected cell × mean fluorescence of background readings).

This CTCF was then compared to the mean CTCF of nearby interphase cells in the same field of view, with the findings shown as a fold increase above interphase values. Three background regions were chosen for each image to correct against autofluorescence.

### 3.9. Statistical Analysis

Data are expressed as the mean of at least four individual observations with the standard deviation (mean ± SD) from three independent experiments (n = 3). Paired Student’s *t*-test analysis was used for the statistical analysis. *p*-values < 0.05 were considered to indicate significance (* *p* < 0.05, ** *p* < 0.01, *** *p* < 0.001).

### 3.10. Molecular Docking

A docking study was performed by Autodock 4.2 [38] to discover the binding mode of the protein and ligands. The X-ray crystal structures of the human monoamine oxidase A in complex with clorgyline (PDB: 2BXR) and human monoamine oxidase B in complex with safinamide (PDB: 2V5Z) were retrieved from the Protein Data Blank database and summited to prepare the receptor wizard. The protein receptor was prepared by the deletion of water molecules and addition of polar hydrogens. The FAD coenzyme was not removed during the docking process. Docking parameter files (gpf and dpf) were generated using the AutoDock tools (ADT) software as setting grid box size of 70/70/70 around center 21.417/2.033/5.763 for MAO-A and 53.317/154.718/26.810 for MAO-B. Docking was performed with 100 runs and a maximum of 2,500,000 evaluations and standard parameters. The protein–ligand complexes and distance measurement (between N5 of FAD and the carbon adjacent to the amine group) were displayed by PyMol [39] to identify the potency of recognition.

## 4. Conclusions

In summary, we developed an AIE fluorogenic probe that undergoes the enzymatic reaction to detect MAO-A and MAO-B inside living cells in a timely manner (within 5 min). The QM-based AIE probe, QM-NH2, was created by combining the substituted phenol with propylamine as a recognition moiety for MAOs. The spectrophotometric analysis revealed that the enzymatic reaction product, QM-OH, had an 8-fold fluorescence enhancement. The HPLC analysis confirmed that QM-OH is the final product after the enzymatic reaction. A series of intracellular imaging experiments of MAOs in cancer cells overexpressing MAO-A (SH-SY5Y) and MAO-B (HepG2) confirms QM-NH2′s ability to detect MAOs in complex biological systems. As QM-NH2 is the first QM-based AIE probe to detect MAOs, we believe that our strategy could pave the way for the development of AIE probes for other enzyme activities.

## Data Availability

Data are contained within the article or Supplementary Materials. Video is available at: https://youtu.be/4eDFGoZFYvU (accessed on 12 March 2023).

## Supplementary Materials

The following supporting information can be downloaded at: https://www.mdpi.com/article/10.3390/molecules28062655/s1. General procedure for the synthesis of QM-OH and QM-NH_2_ and compound characterizations; Figure S1: Fluorescence response of **QM-NH_2_** to MAOs in the presence of interferences; Figure S2: Effects of temperature on the fluorescence intensity of **QM-NH_2_** and **QM-OH**, Figure S3: Stability of **QM-NH_2_** and **QM-OH** in HEPES buffer; Figure S4: Confocal images of time-dependent enzymatic reaction detection of **QM-NH_2_** in SH-SY5Y and HepG2 cells; Figure S5: Confocal images of SH-SY5Y, HepG2, and HFF cells treated with **QM-NH_2_** for 1–2 h; Figure S6: Confocal images of various cancer cells treated with **QM-NH_2_** for 3 h.; Table S1: Docking results.

## Sample Availability

Samples of **QM-OH** and **QM-NH_2_** are available from the authors.
