# Supplementary material for: Quinoline-Malononitrile-Based Aggregation-Induced Emission Probe for Monoamine Oxidase Detection in Living Cells
Source: Molecules. 2023 Mar 15;28(6):2655. doi: 10.3390/molecules28062655 (PMC10054884; doi:10.3390/molecules28062655)
Supplement: Supplementary file 1 [file molecules-28-02655-s001.zip › molecules-2238390-supplementary.pdf]

Supporting Information

Quinoline-Malononitrile-Based Aggregation-Induced Emission Probe for Monoamine Oxidase Detection in Living Cells

Chuthamat Duangkamol [1,2], Sirilak Wangngae [1], Sirawit Wet-osot [3], Onnicha Khaikate [1], Kantapat Chansaenpak [4], Rung-Yi Lai [1,*] and Anyanee Kamkaew [1,*]

[1] School of Chemistry, Institute of Science, Suranaree University of Technology,

Nakhon Ratchasima 30000, Thailand

[2] Division of Basic and Medical Sciences, Faculty of Allied Health Sciences, Pathumthani University, Pathum Thani 12000, Thailand

[3] Medical Life Science Institute, Department of Medical Sciences, Ministry of Public Health,

Nonthaburi 11000, Thailand; sirawitwetosot@gmail.com

[4] National Nanotechnology Center, National Science and Technology Development Agency, Thailand Science Park, Pathum Thani 12120, Thailand; kantapat.cha@nanotec.or.th

* Correspondence: rylai@sut.ac.th (R.-Y.L.); anyanee@sut.ac.th (A.K.)

Experimental Section

Materials and instruments

All glassware was oven-dried prior to use. All the reagents were purchased from commercial sources (Sigma Aldrich, TCI, Carlo Erba, Acros, and Merck) and used without further purification. Thin layer chromatography (TLC) was performed using silica gel 60 F254 (Merck) and visualized using UV light. Column chromatography was performed with silica gel (mesh 300-400). ^{1}H NMR and ^{13}C NMR spectra were recorded on a Bruker Avance 500 MHz spectrometer at room temperature in DMSO-d_6 with Me_4Si as an internal standard. Chemical shifts of ^{1}H NMR spectra were recorded and reported in ppm from the solvent resonance (DMSO-d_6 at 2.49 ppm). Data were reported as follows: a chemical shift in ppm (δ), multiplicity (s = singlet, d = doublet, t = triplet, q = quartet, quin = quintet, br = broad, and m = multiplet), coupling constant in hertz (Hz) and integration and only major peaks are reported in cm^{-1}. ^{13}C NMR spectra were also recorded in ppm from the solvent resonance (DMSO-d_6 at 39.52 ppm). HRMS and mass data were recorded by ESI on a TOF mass spectrometer.

General spectral analysis for MAO

The stock solutions (1 mM) of probes were prepared in DMSO, which was diluted with hydroxyethyl piperazineethanesulfonic acid (HEPES) buffer (100mM HEPES, pH=7.4 with 5% glycerol and 1% DMSO) to 10 μM for absorbance or fluorescence spectroscopic measurements. The absorbance and fluorescence spectrum changes of the reaction system (HEPES/DMSO = 9:1 v:v, pH = 7.4, 37 °C) were measured using an ultraviolet analyzer and a fluorometer after the addition of an appropriate volume of MAO (MAO-A or MAO-B) and/or other analytes (excitation wavelength: 445 nm, excitation and emission slit widths: 10 nm). The data are presented with three different measurements' mean standard deviation (SD).

Synthesis of probe QM-NH_2

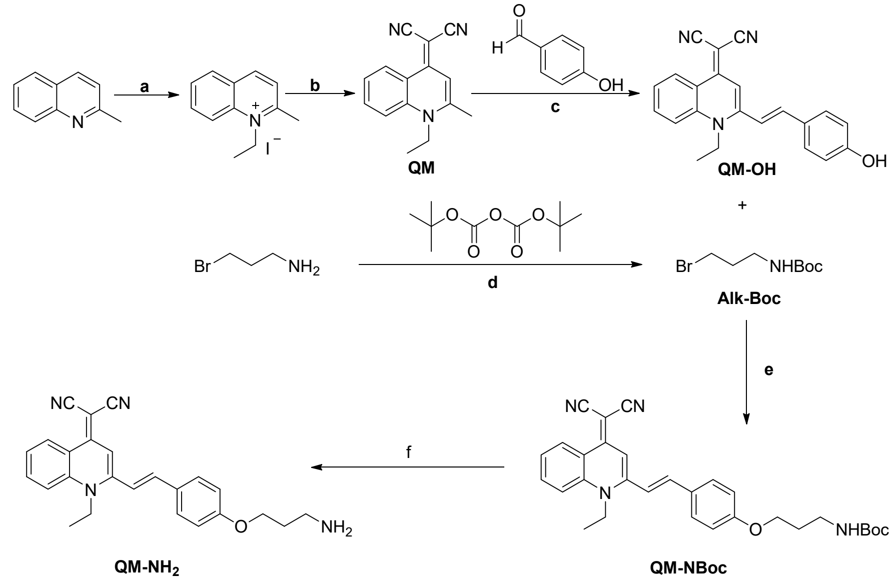

Scheme S1. Synthesis of probe **QM-NH_2**. Reagents and conditions: a) Iodoethane, CH_3CN, reflux for 12 h; b) Malononitrile, piperidine, Na, EtOH, 0 ºC, 4h; c) Piperidine, CH_3CN, reflux for 1 h; d) Et_3N, DCM, rt, overnight; e) K_2CO_3, DMF, rt, overnight; f) TFA, dry CH_2Cl_2, 0 ºC, overnight.

Preparation of QM-OH. Quinoline-malononitrile (**QM**, 1 g, 4.25 mmol) and 4-hydroxybenzaldehyde (623 mg, 5.11 mmol) were dissolved in 30 mL of acetonitrile with piperidine (1.0 mL) under a nitrogen atmosphere at room temperature. The mixture was then refluxed for 12 h. The crude product was collected by filtration, followed by recrystallization to afford the desired product **QM-OH** (822 mg): yield 57%.[1]

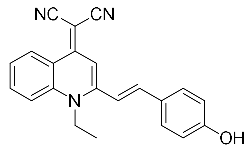

(*E*)-2-(1-ethyl-2-(4-hydroxystyryl)quinolin-4(1*H*)-ylidene)malononitrile. Characterization of **(QM-OH)**; HRMS: m/z $[M+Na]^+$ calc for $C_{22}H_{17}N_3NaO^+$: 362.1264; found: 362.1264. ^{1}H NMR (500 MHz, DMSO-d_6) δ 10.07 (s, 1H), 8.99 (d, J = 9.0 Hz, 1H), 8.14 (d, J = 9.0 Hz, 1H), 7.98 (t, J = 7.0 Hz, 1H), 7.74 (d, J = 8.5 Hz, 2H), 7.66 (t, J = 8.0 Hz, 1H), 7.40 (quartet, J = 16.0 Hz, 2H), 7.08 (s, 1H), 6.93 (d, J = 9.0 Hz, 2H), 4.64 (quartet, J = 7.5 Hz, 2H), 1.50 (t, J = 6.5 Hz, 3H), ^{13}C NMR (125 MHz, DMSO-d_6) δ 159.9, 152.6, 150.0, 140.4, 138.3, 134.0, 130.4, 126.8, 125.7, 125.2, 121.1, 118.4, 117.3, 116.2, 107.0, 47.1, 44.2, 14.1.

Preparation of QM-NBoc. A solution of compound **QM-OH** (0.200 g, 0.59 mmol), *t*-Boc-protected primary bromopropylamine **(Alk-Boc),** and K_2CO_3 (0.122g, 0.89 mmol) in DMF (5mL) was stirred at room temperature for overnight. After the removal of the organic solvent, the product was purified by column chromatography using 60% EtOAc/Hexane as the eluent in a yield of 68%.

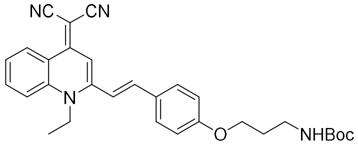

Tert-butyl (*E*)-(3-(4-(2-(4-(dicyanomethylene)-1-ethyl-1,4-dihydroquinolin-2-yl)vinyl) phenoxy)propyl)carbamate, Characterization of **QM-NBoc**; HRMS: m/z $[M+Na]^+$ calc for $C_{30}H_{32}N_4NaO_3^+$: 519.2366; found: 519.2367. ^{1}H NMR (500 MHz, DMSO-d_6) δ 9.00 (d, *J* = 8.5 Hz, 1H), 8.16 (d, *J* = 9.0 Hz, 1H), 8.00 (t, *J* = 8.5 Hz, 1H), 7.85 (d, *J* = 9.0 Hz, 2H), 7.69 (t, *J* = 7.5 Hz, 1H), 7.47 (s, 2H), 7.09 (d, *J* = 2.5 Hz, 2H), 7.07 (s, 1H), 4.65 (quartet, *J* = 7.0 Hz, 2H), 4.12 (t, *J* = 6.0 Hz, 2H), 3.17 (quartet, *J* = 6.0 Hz, 2H), 2.58 (s, 9H), 1.93 (quintet, *J* = 6.5 Hz, 2H), 1.48 (t, *J* = 7.0 Hz, 3H), ^{13}C NMR (125 MHz, DMSO-d_6) δ 160.6, 156.1, 152.7, 150.0, 140.0, 138.3, 134.2, 130.3, 128.3, 125.6, 125.4, 121.1, 118.6, 118.5, 115.3, 107.1, 78.0, 66.0, 47.0, 44.3, 37.4, 29.6, 28.7, 14.1.

Preparation of QM-NH$_2$. The compound **QM-NBoc** (0.150 g, 0.30 mmol) was added to dry dichloromethane (20 mL) containing 10% (v/v) TFA. The resulting mixture was stirred at 0 ºC overnight. After the solvent was removed by evaporation, the product was purified by flash chromatography using 1–5% MeOH/CH_2Cl_2 as the eluent to give **QM-NH$_2$** (0.67 g, 56% yield) as an orange solid.

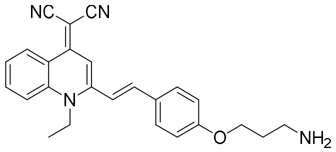

(*E*)-2-(2-(4-(3-aminopropoxy)styryl)-1-ethylquinolin-4(1*H*)-ylidene)malononitrile,
Characterization of **QM-NH$_2$**; HRMS: m/z $[M+Na]^+$ calc for $C_{25}H_{25}N_4O^+$: 397.1950; found: 397.2023. ^{1}H NMR (500 MHz, DMSO-d_6) δ 8.99 (d, *J* = 8.5 Hz, 1H), 8.23 (s, 2H), 8.15 (d, *J* = 9.0 Hz, 1H), 7.99 (t, *J* = 7.5 Hz, 1H), 7.85 (d, *J* = 9.0 Hz, 2H), 7.66 (t, *J* = 7.5 Hz, 1H), 7.47 (s, 2H), 7.09 (d, *J* = 8.5 Hz, 2H), 7.08 (s, 1H), 4.64 (quartet, *J* = 7.5 Hz, 2H), 4.20 (t, *J* = 6.0 Hz, 2H), 3.06 (s, 2H), 2.16 (s, 2H), 1.48 (t, *J* = 7.0 Hz, 3H), ^{13}C NMR (125 MHz, DMSO-d_6) δ 160.2, 152.7, 149.9, 139.9, 138.3, 134.2, 130.3, 128.4, 125.6, 125.4, 121.1, 121.1, 118.8, 118.6, 118.5, 116.4, 115.3, 114.0, 107.1, 65.29, 47.0, 44.3, 36.7, 27.2, 14.1.

^{1}H and ^{13}C NMR Spectra

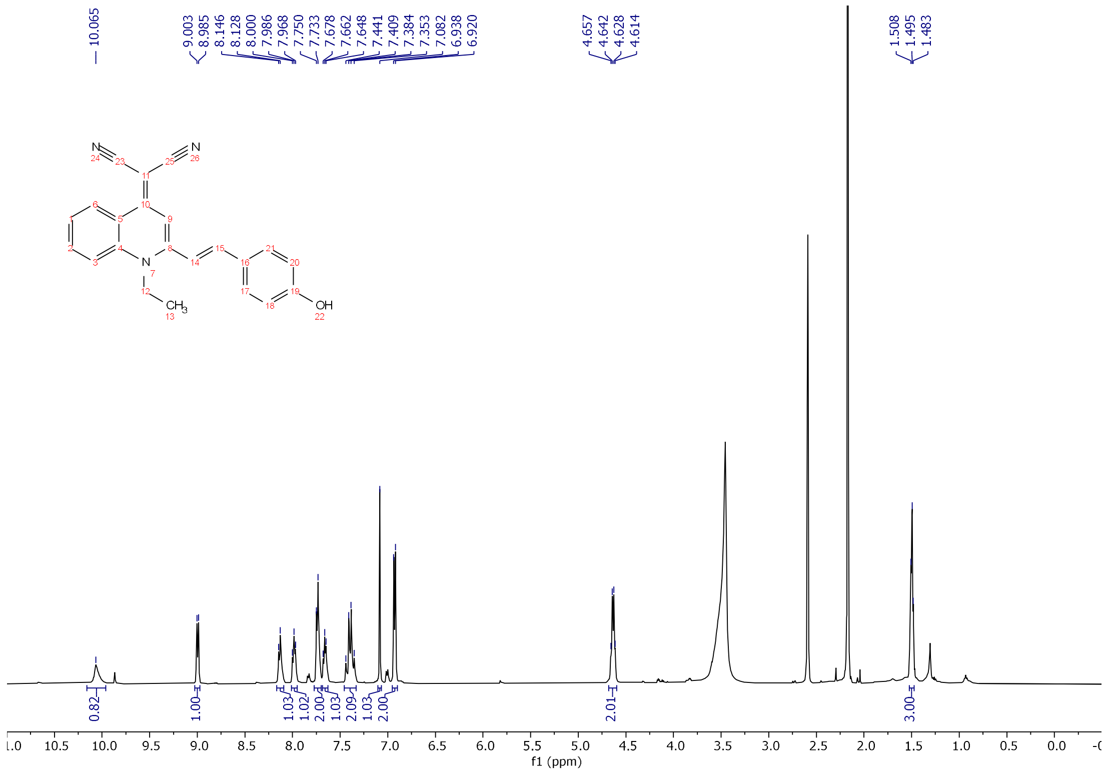

^{1}H NMR of compound QM-OH

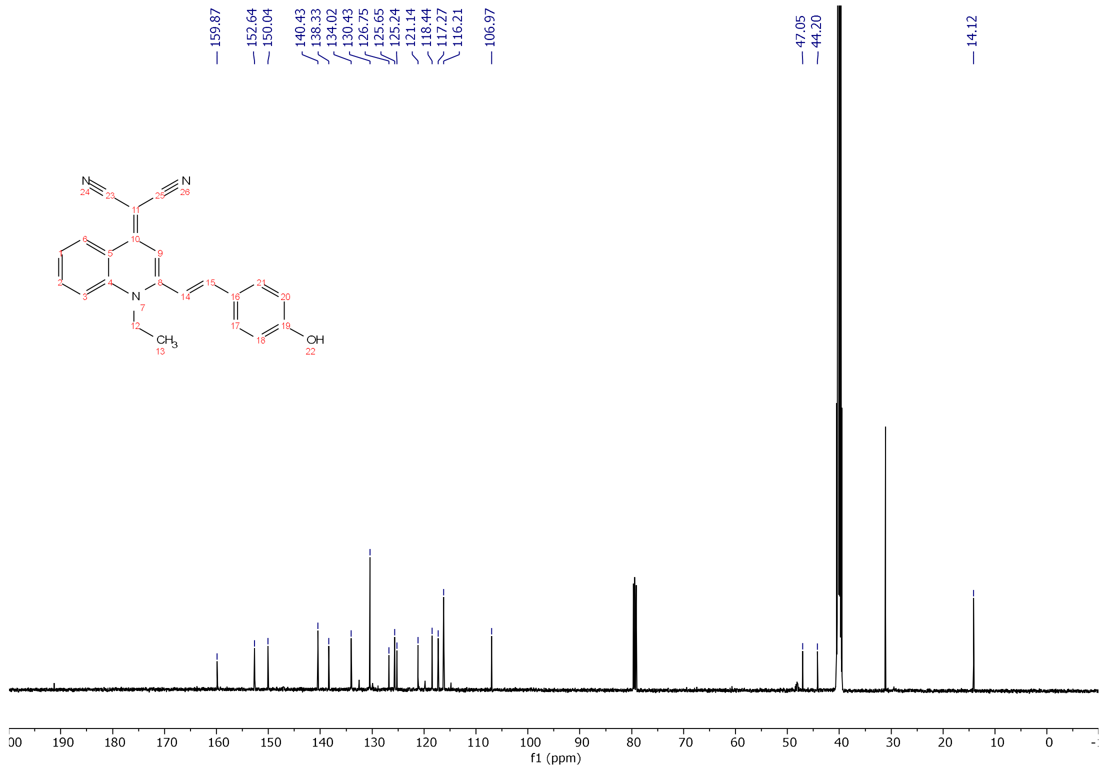

^{13}C NMR of compound QM-OH

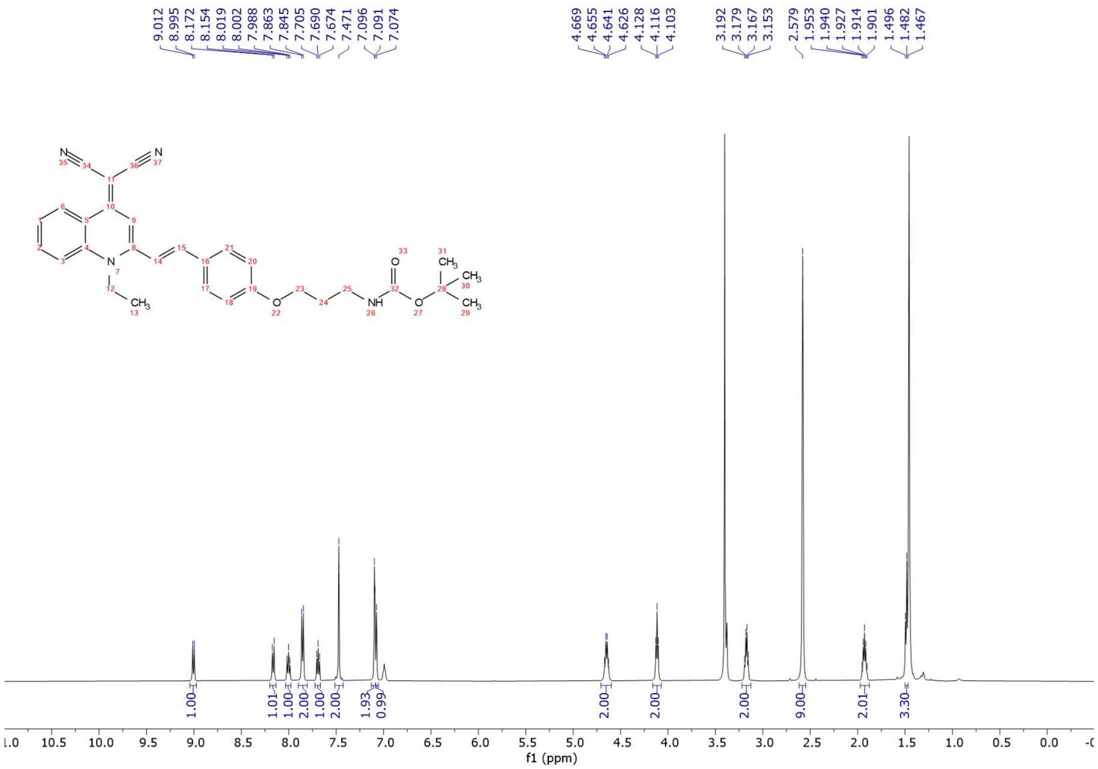

^{1}H NMR of compound QM-NBoc

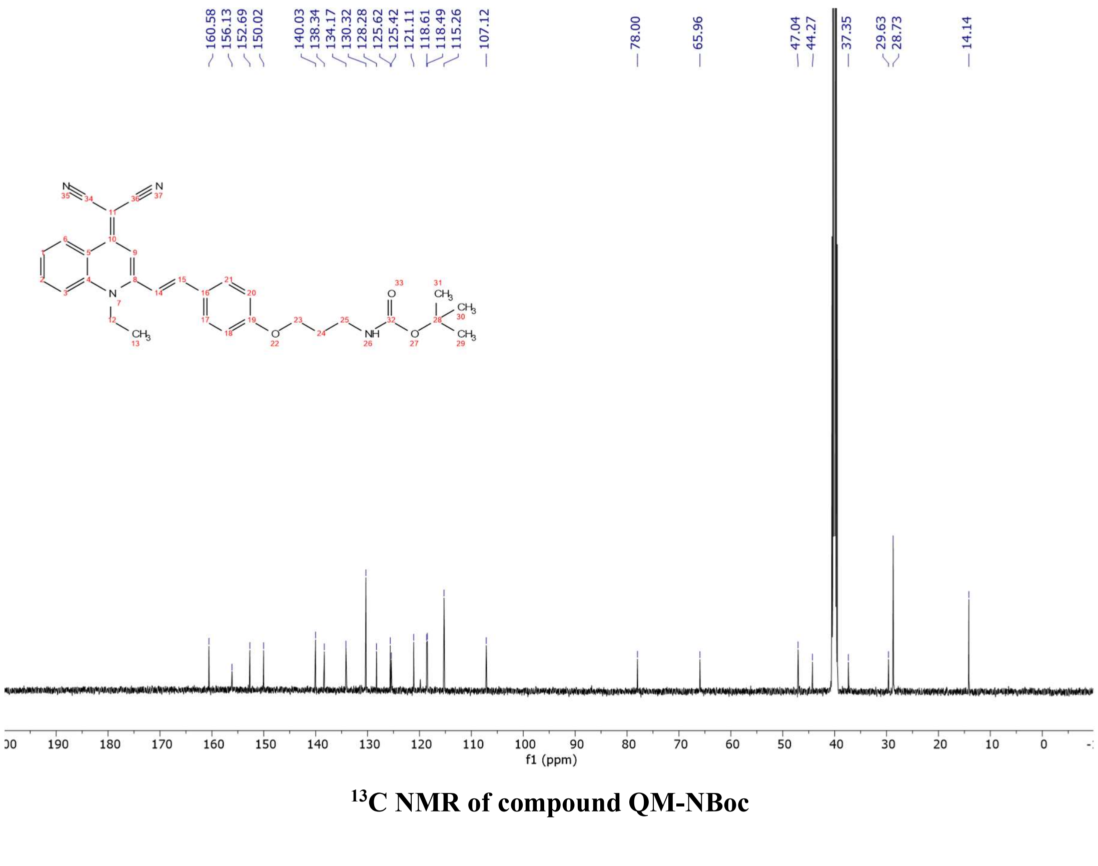

^{13}C NMR of compound QM-NBoc

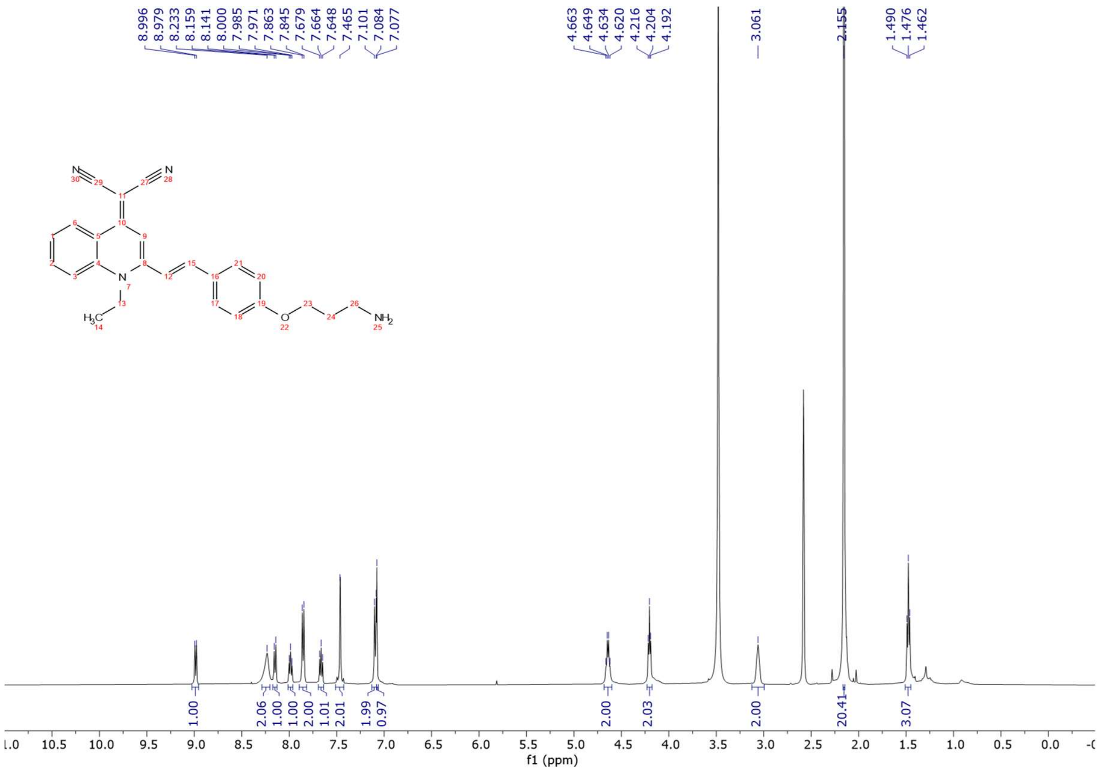

^{1}H NMR of compound QM-NH_2

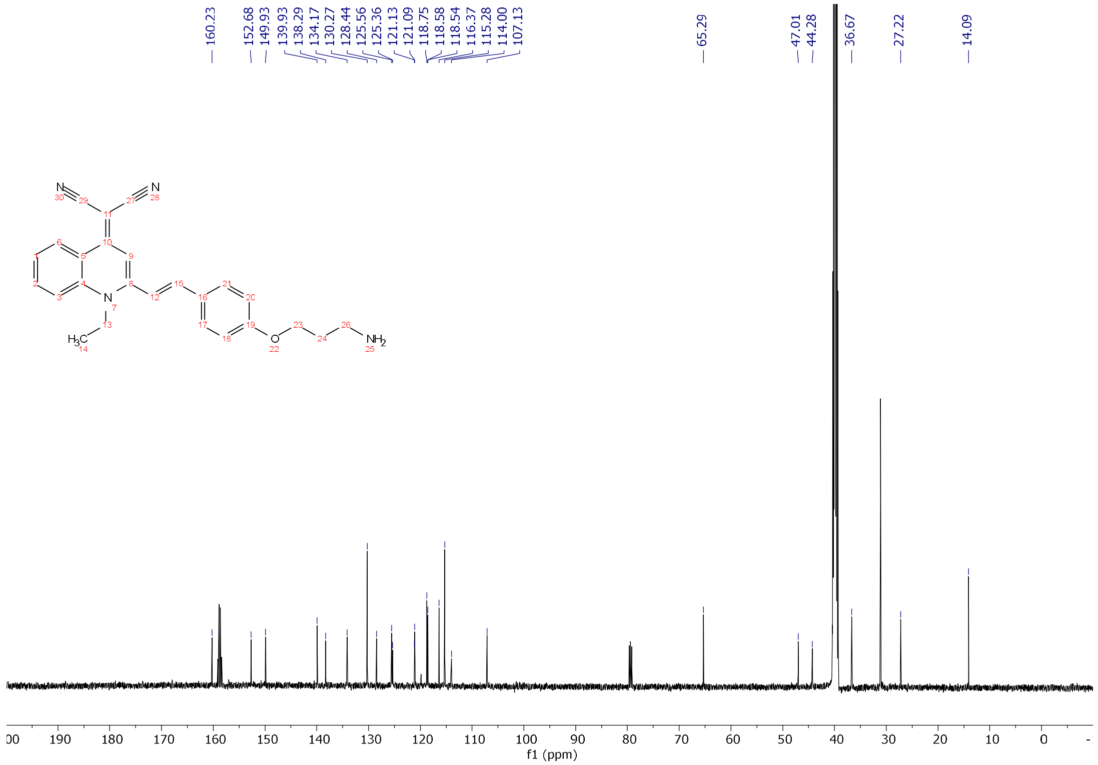

^{13}C NMR of compound QM-NH_2

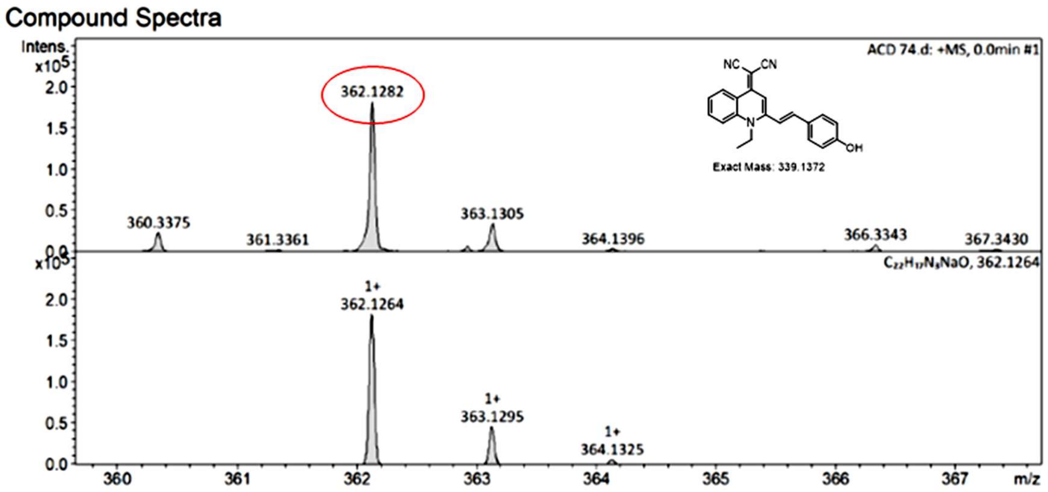

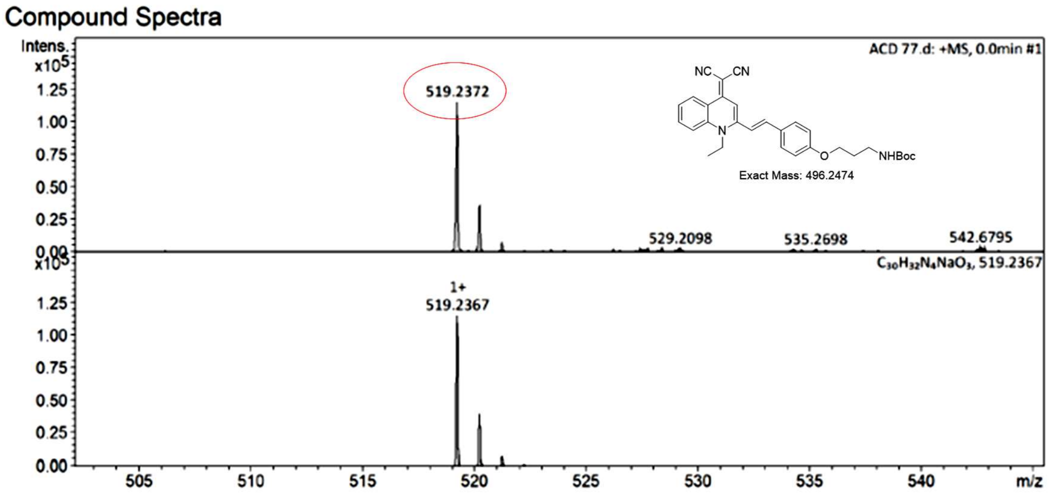

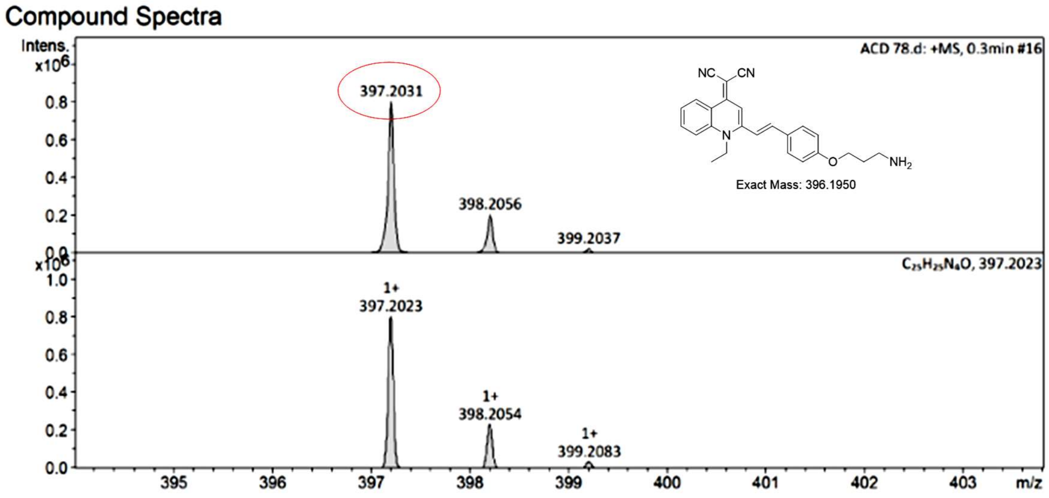

ESI mass spectra of the reaction solutions of probe **QM-R**.

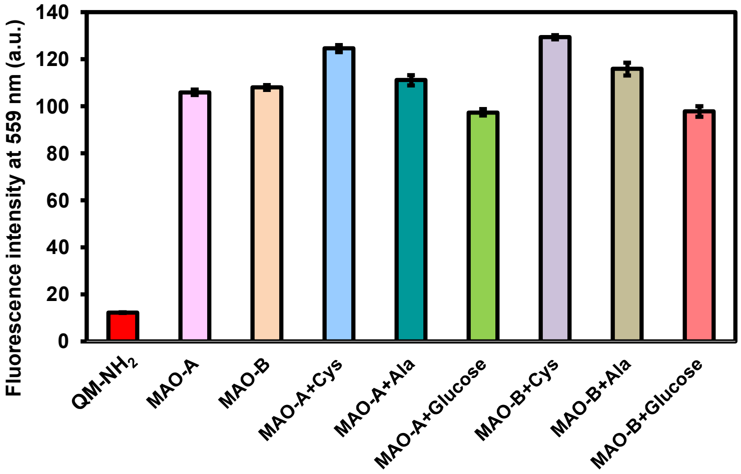

Figure S1. Fluorescence response of **QM-NH₂** (10 µM) after 5 min of incubation at 37 ºC with MAO-A (20 µg/mL) or MAO-B (20 µg/mL) in the presence of high concentrations of oxidizable interferences such as amino acids (alanine and cysteine, 1 mM) and glucose (10 mM).

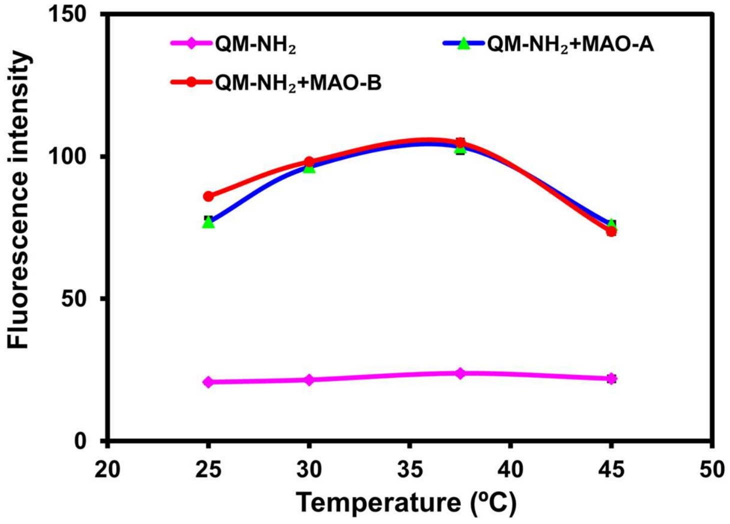

Figure S2. Effects of temperature on the fluorescence intensity of **QM-NH₂** (pink), **QM-OH** (blue), **QM-NH₂** (10 µM) with MAO-A (20 µg/mL) (green) and **QM-NH₂** (10 µM) with MAO-B (20 µg/mL) (red) in HEPES buffer pH 7.4, monitored at 559 nm and λ_{ex} = 445 nm. The results are expressed as the mean SD (n = 3).

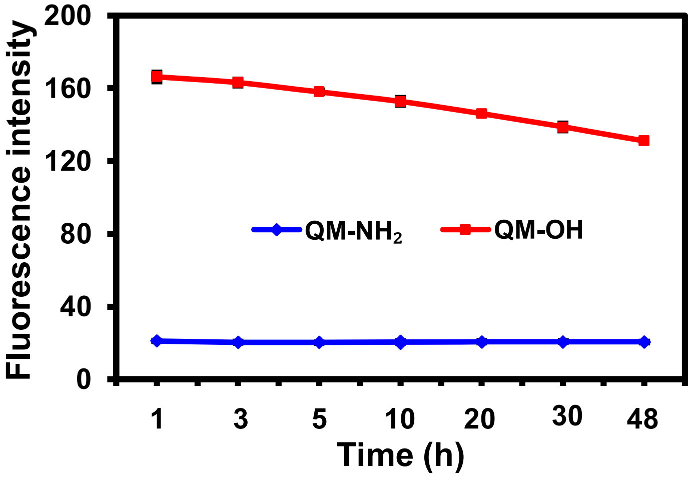

Figure S3. Stability of **QM-NH$_2$** and **QM-OH** (10 μM) in HEPES buffer investigated by determining its fluorescence intensity changes, monitored at 559 nm and λ_{ex} = 445 nm. The results are expressed as the mean SD (n = 3).

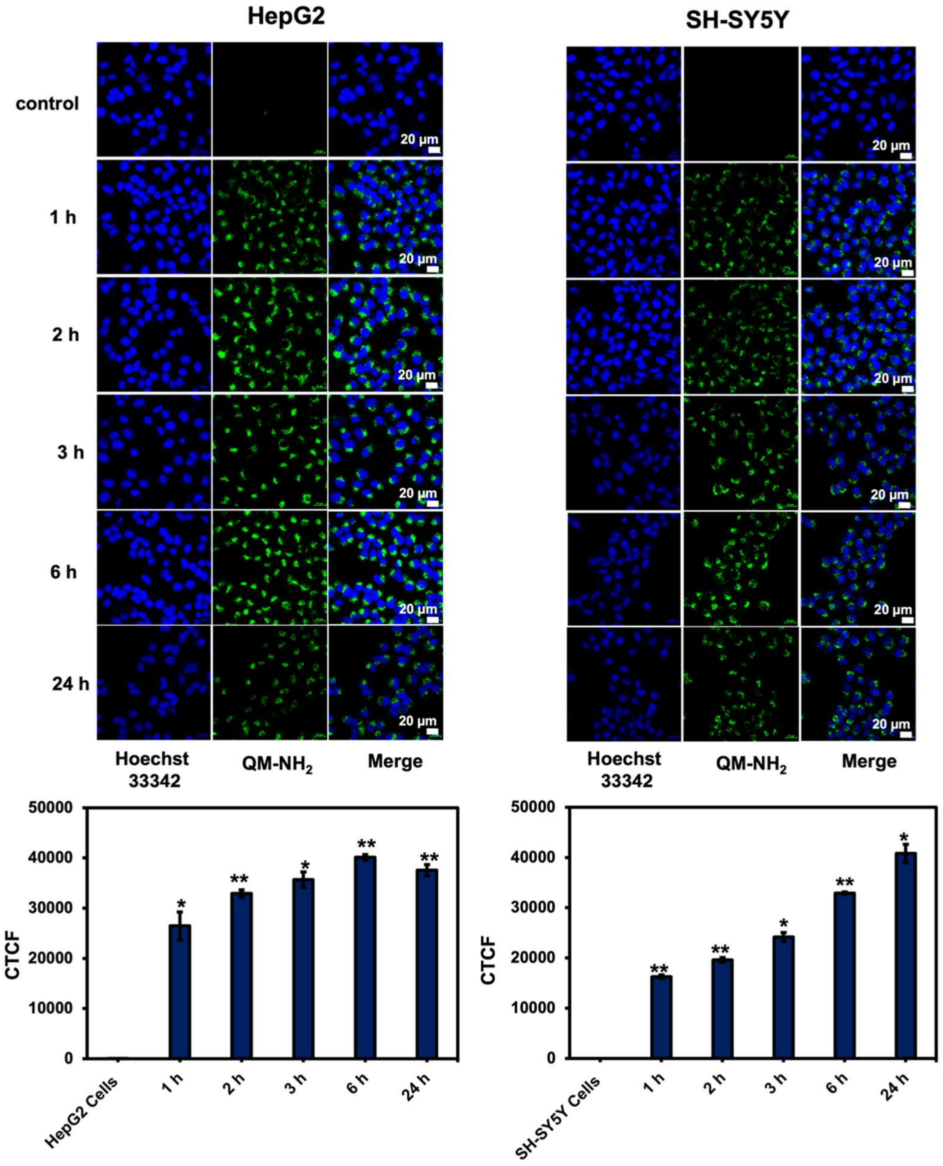

Figure S4. Confocal images of SH-SY5Y and HepG2 live cells incubated with 10 μM of **$QM-NH_2$** for 1 to 24 h were obtained using a laser scanning confocal microscope (Nikon A1Rsi, 63× oil immersed optics) and quantitative fluorescent intensity represented as corrected total cell fluorescence (CTCF), which were quantified using ImageJ and represent the mean ± SD (from three independent experiments, 30 cells/set, respectively. Scale bar = 20 μm. Nucleus is shown in blue as Hoechst 33342 signal (excitation laser = 405 nm, emission band 450/25 nm), **QM-OH** can be detected as green fluorescence (excitation laser = 488 nm, emission band 515/30 nm).

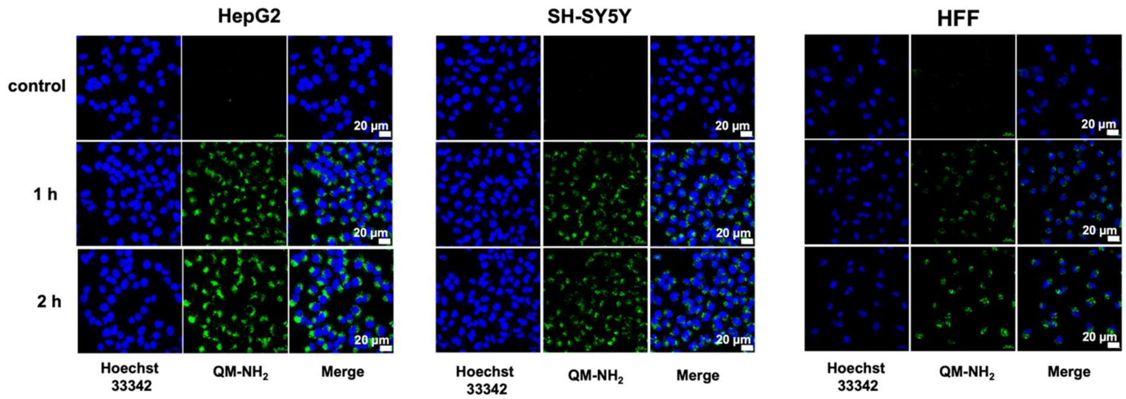

Figure S5. Confocal images of SH-SY5Y, HepG2, and HFF cells treated with 10 μM of **QM-NH₂** for 1-2 h.

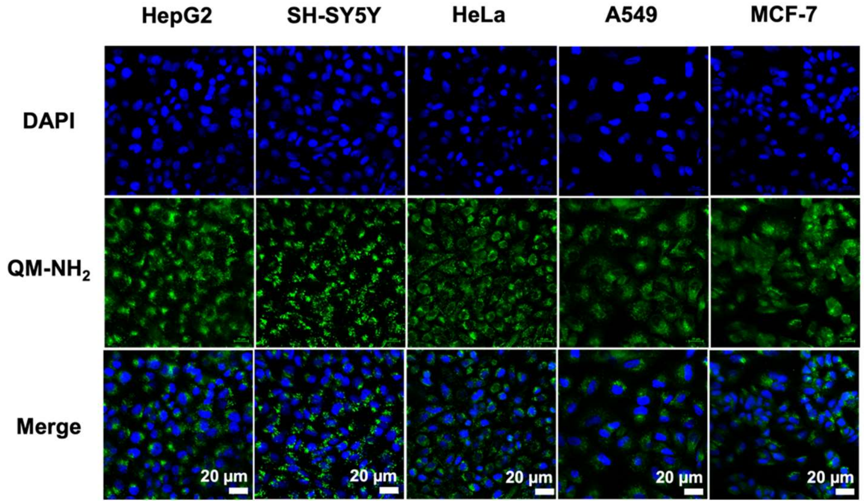

Figure S6. Confocal images of various cancer cells treated with 10 μM of **QM-NH₂** for 3 h.

Table S1. Docking results

Molecule name	Binding Energy kcal/mol	No. of H-bond	H-bond interacting residue	Hydrophobic interaction
MAO-A (PDB:2BXR)				
QM-NH_2	-9.66	2	Asn181, Met324	Leu97, Phe208, Ile335, Leu337, Tyr407, Tyr444
Tyramine	-4.77	2	Glu216, Thr336	Phe208, Ile335, Leu337
MAO-B (PDB:2V5Z)				
QM-NH_2	-12.65	2	Cys172, Thr201, Tyr326	Trp119, Leu164, Phe168, Leu171, Ile199, Gln206. Tyr326, Phe343, Tyr398, Tyr435
Tyramine	-5.40	2	Cys172, Ile199	Leu171, Ile199, Tyr326

References

1. Wang, M.; Yang, N.; Guo, Z.; Gu, K.; Shao, A.; Zhu, W.; Xu, Y.; Wang, J.; Prud'homme, R. K.; Guo, X., Facile preparation of AIE-active fluorescent nanoparticles through flash nanoprecipitation. *Industrial & Engineering Chemistry Research* **2015,** *54* (17), 4683-4688.
